# Maternal Residential Proximity to Major Roadways and the Risk of Childhood Acute Leukemia: A Population-Based Case-Control Study in Texas, 1995–2011

**DOI:** 10.3390/ijerph16112029

**Published:** 2019-06-07

**Authors:** Erin C. Peckham-Gregory, Minh Ton, Karen R. Rabin, Heather E. Danysh, Michael E. Scheurer, Philip J. Lupo

**Affiliations:** 1 Department of Pediatrics, Section of Hematology-Oncology, Baylor College of Medicine, One Baylor Plaza, MS: BCM622, Houston, TX 77030, USA; Erin.Peckham@bcm.edu (E.C.P.-G.); Krrabin@txch.org (K.R.R.); Hdanysh@rti.org (H.E.D.); Scheurer@bcm.edu (M.E.S.); 2 Texas Children’s Cancer and Hematology Centers, Texas Children’s Hospital, Feigin Center, 1102 Bates St, Houston, TX 77030, USA; 3 Department of Economics, Martel College, Rice University, 99 Sunset Blvd, Houston, TX 77005, USA; Mdt3@rice.edu

**Keywords:** epidemiology, acute leukemia, traffic-related air pollution, Texas Cancer Registry

## Abstract

Acute leukemia is the most common pediatric malignancy. Some studies suggest early-life exposures to air pollution increase risk of childhood leukemia. Therefore, we explored the association between maternal residential proximity to major roadways and risk of acute lymphoblastic leukemia (ALL) and acute myeloid leukemia (AML). Information on cases with acute leukemia (n = 2030) was obtained for the period 1995–2011 from the Texas Cancer Registry. Birth certificate controls were frequency matched (10:1) on birth year (n = 20,300). Three residential proximity measures were assessed: (1) distance to nearest major roadway, (2) residence within 500 meters of a major roadway, and (3) roadway density. Multivariate logistic regression was used to generate adjusted odds ratios (aOR) and 95% confidence intervals (CI). Mothers who lived ≤500 meters to a major roadway were not more likely to have a child who developed ALL (OR = 1.03; 95% CI: 0.91–1.16) or AML (OR = 0.84; 95% CI: 0.64–1.11). Mothers who lived in areas characterized by high roadway density were not more likely to have children who developed ALL (OR = 1.06, 95% CI: 0.93–1.20) or AML (OR = 0.83, 95% CI: 0.61–1.13). Our results do not support the hypothesis that maternal proximity to major roadways is strongly associated with childhood acute leukemia. Future assessments evaluating the role of early-life exposure to environmental factors on acute leukemia risk should explore novel methods for directly measuring exposures during relevant periods of development.

## 1. Introduction

Acute leukemia is the most common childhood cancer in the United States and consists of two main subtypes: acute lymphoblastic leukemia (ALL; 73% of cases ages 0–19 years) and acute myeloid leukemia (AML; 18% of cases ages 0–19 years) [1]. Due to advances in therapy, 5-year survival for those diagnosed with acute leukemia is approaching 90% [2,3,4]. However, survivors often face chronic health conditions as a consequence of their therapy, including diabetes mellitus, cardiovascular disease, and neurocognitive deficits [5,6]. Additionally, the incidence of childhood leukemia has been steadily increasing since the 1970s [7,8]. Despite this, very little is known about the causes of acute leukemia, which severely limits prevention and surveillance efforts. In fact, outside of some confirmed risk factors, like cancer predisposition syndromes and exposure to ionizing radiation, over 90% of cases are of unknown etiology [9].

There are growing public health concerns over the role of ambient air pollution exposure on the risk of developing cancer, both in children and adults [10,11,12]. This is in part due to known and suspected carcinogenic compounds (e.g., benzene) that are present in motor vehicle emissions [13]. Additionally, several studies have established that those who live near major roadways or areas of high traffic density have higher levels of air pollution exposure compared to those who do not live near major roadways [13,14,15]. Several proximity measures have been used to study the association between distance to traffic and risk of cancers, including leukemia [16,17]. These distance to traffic measures include distance to nearest major roadway, buffer distances to major roadways, and measures of roadway density (total km of roads within a buffer) and/or vehicle density [18,19,20]. Studies have suggested that the closer one lives in proximity to a major roadway, the greater the increase in risk of developing cancer and other comorbidities [21,22,23]. This is notable as approximately 40% of individuals in the United States live near a major roadway, and this proportion continues to grow [13].

Some studies have suggested that exposure to motor vehicle emissions is associated with the risk of childhood cancer, including acute leukemia [24,25,26,27]. However, to date, results have been equivocal, which in part may be due to differences in exposure levels across geographic regions, exposure assessment methods, small sample sizes, and case inclusion criteria. Because of the increasing incidence of acute leukemia, the growing proportion of the population living in areas characterized by higher levels of ambient air pollution, and inconclusive results in previous assessments, we leveraged one of the largest and most diverse population-based cancer registries in the world in a geographical region characterized by high and variable levels of air pollution to evaluate the role of early-life exposure to ambient air pollution on the risk of developing acute leukemia.

## 2. Materials and Methods

### 2.1. Study Design and Population

To assess the association between maternal residential proximity to major roadways and risk of childhood leukemia, we conducted a population-based case-control study. Information on cases was obtained from the Texas Cancer Registry (TCR), a statewide population-based cancer registry administered by the Texas Department of State Health Services (TX DSHS), which was Gold certified by the North American Association of Central Cancer Registries during the study period. Specifically, the TCR identified children born in Texas between 1 January 1995 and 31 December 2011 who were diagnosed with an acute leukemia before 17 years of age during the same period. We used the *International Classification of Childhood Cancer, Third Edition* (ICCC-3) to define the acute leukemia subtypes: ALL (ICCC-3 group Ia) and AML (ICCC-3 group Ib) [28]. Only cases diagnosed with acute leukemia as their first primary malignancy were included. For each case, the TCR also provided information on age at the time of diagnosis and used probabilistic linkage to link the cancer registry record to Texas birth certificates.

Control subjects were randomly selected among the birth certificate records of those who were both born in Texas during the same period as cases (1995–2011) and were confirmed not to be included in the TCR. Controls were frequency matched to cases on birth year at a ratio of ten control subjects for every acute leukemia case. For all study subjects, we obtained the geocoded address of the maternal residence at the time of delivery (i.e., at the time of the child’s birth), as well as other child- and maternal-related information, from the birth certificate records.

### 2.2. Exposure Assessment

We determined maternal residential proximity to major roadways using the Texas roadway network StratMap. StratMap was obtained from the Texas National Resources Information System, which maintains the largest collection of geographic data for Texas [29]. In assessing exposure to traffic-related air pollution, we utilized three proxy measures based on maternal residential proximity to major roadways, which are described in detail below: (1) continuous distance of the residence to the nearest major roadway, (2) the presence of a major roadway within a 500 meter (m) radius of the residence, and (3) the density of major roadways within a 500 m radius of the residence [19]. We selected a buffer of 500 m, as the area of 500 m adjacent to a major roadway has been found to have the highest concentration of primary air pollutants from traffic emissions and was designated as a buffer region to assess exposure to air pollution [13]. Roadways defined by the U.S. Census Bureau as interstate, state, county, or toll highways (with an A1, A2, or A3 Feature Class Code) were designated as a major roadway [30]. The maternal residential address listed on each subject’s birth certificate was geocoded by the TX DSHS Center for Health Statistics. The longitude and latitude data points from the geocoded addresses established residential location and distance to major roadways.

A geographic information systems (GIS) approach was used to assess exposure and geocode street addresses according to 2007 Tiger/Line files [30]. ArcGIS (version 10.0, Environmental Systems Research Institute Inc., Redlands, CA, USA) was used for spatial analyses. We used the Euclidian distance as the distance of the maternal residence to the nearest side of the closest major roadway. This distance was calculated as a continuous measure in meters. Buffer analyses consisted of determining if a major roadway was within a 500 m radius from maternal residence. Exposure was determined based on the presence or absence of a major roadway within the 500 m radius around the maternal residence. Roadway density was determined by summing all major roadway segments (km) that fell within the 500 m radius of the maternal residence and then modeled as a categorical variable. In other words, roadway density refers to the actual amount of km of major roadway segments within the 500 m radius of maternal residence. The “low roadway density” category included mothers living outside the 500 m buffer radius from the nearest major roadway, while mothers residing within 500 m of a major roadway were defined as residing in an area with “medium roadway density” or “high roadway density” based on the total length of major roadway within the 500 m buffer. Consistent with previous studies [19,31], the cutoff between medium and high density (2.11 km) was determined using the median split of the distribution of roadway density among the control subjects living within 500 m of a major roadway.

### 2.3. Covariate Selection

Covariates selected for the current analysis included child’s sex, age at diagnosis, season of birth, gestational size, term of birth (<37 weeks, 37–40, 41–42, and ≥42), maternal self-reported race/ethnicity (non-Hispanic white, non-Hispanic black, Hispanic, non-Hispanic other), maternal age at time of delivery (<25, 25–30, 31–35, and ≥35 years), maternal education (<high school, high school, or >high school), urban versus rural status of residence, and area-level poverty [32,33,34,35,36,37,38,39]. We determined season of birth by categorizing every child’s birth date as spring, summer, fall, or winter based on solstice and equinox dates. The date of onset of last menstrual period (LMP) and the clinical estimate of gestation in weeks were obtained from birth certificates for cases and controls. For most births, gestational age was established using the LMP [40,41]. Based on previous literature, we utilized the clinical estimate as a substitute for LMP if the LMP date was missing or if the absolute difference between the LMP date and clinical estimate was greater than 14 days [41,42,43]. The birth was excluded from analyses of birth term or size-for-gestational-age if both the LMP date and the clinical estimate were missing. Births were categorized as small-for-gestational-age if the birth weight was <10th percentile for their gestational age or large-for-gestational-age if the birth weight was >90th percentile for their gestational age. The 2009–2010 U.S. live birth data stratified by infant sex provided data for the gestational estimates and percentiles [44].

Information on covariates was obtained from the child’s birth certificate. Additionally, area-level poverty was used as a surrogate for socioeconomic status (SES). We obtained information from the 2000 US Census on the proportion of households with an income lower than the poverty level in a census tract (a small statistical subdivision of a county designed to represent a relatively homogenous group of approximately 1200 to 8000 people) [45]. Census tracts where ≥15% of households had an income above the poverty level were classified as a high area-level poverty region while census tracts where <15% of households had an annual income below the poverty level were classified as a low area-level poverty region.

### 2.4. Statistical Analysis

We abstracted descriptive statistics on child and maternal demographic characteristics along with area-level factors, such as poverty and urban status, for both cases and controls. The distribution of the exposure (e.g., means and frequencies) among cases and controls was calculated for each of the residential proximity measures. *p*-values comparing variables between cases and controls were generated through chi-squared tests for categorical variables or t-tests for continuous variables. Multivariate unconditional logistic regression was used to assess the association between maternal residential proximity to major roadways and each acute leukemia subtype. We conducted these analyses overall (ages 0–16 years) and among those ≥1 to ≤5 years of age at diagnosis and less than one year of age at diagnosis to reflect differences in cytogenetic profiles among younger cases [46,47], as well as the potential for stronger perinatal exposure effects among those diagnosed earlier in life [25]. We generated unadjusted and adjusted odds ratios (OR and aOR, respectively) along with 95% confidence intervals (CI) to assess the association between each exposure variable of interest and acute leukemia subtypes. Covariates in the adjusted models were selected a priori based on previous literature for the specific acute leukemia subtype. For mothers who had children diagnosed with either ALL or AML, we considered the covariates of race/ethnicity (reference = non-Hispanic white), education level (reference = completed high school), socioeconomic status (reference = low-area level poverty), birth weight in grams (continuous), and child sex (reference = male), along with the matching factor of birth year. Factors such as race/ethnicity [48], education [49], and SES [50] have been shown to be potential confounders in the context of ambient air pollution exposure. We additionally considered the covariate of birth weight, since high birth weight is associated with ALL [33,35,51]. Birth year was included as a covariate in all regression models since this was the matching factor for cases and controls. All statistical analyses were conducted using Stata, version 14.2 (StataCorp LP, College Station, TX, USA).

## 3. Results

Maternal and perinatal characteristics of acute leukemia cases born in Texas and diagnosed between 1995 and 2011 are shown in Table 1. Of the 2030 acute leukemia cases included in this study, the majority of cases were ALL (86.0%), while the remaining 14.0% were AML. Regardless of acute leukemia subtype, most children were in the age group of ≥1 to ≤5 years (67.8%), consistent with known childhood incidence rates [1]. In this study, almost all mothers lived in urban areas (95.8%). Compared to controls, a higher proportion of cases were male (54.9% of cases vs. 50.5% controls; *p*-value = 0.001), of large gestational size (11.6% of cases vs. 8.4% controls; *p*-value < 0.001), and of Hispanic ethnicity (53.5% of cases vs. 43.2% of controls; *p*-value < 0.001). Additionally, at the time of delivery, mothers with children diagnosed with acute leukemia were slightly more likely to be at least 35 years of age (14.2% of case mothers vs. 10.7% of control mothers; *p*-value < 0.001) and not have a high school diploma (33.6% of case mothers vs. 29.8% of control mothers; *p*-value = 0.001). Relative to controls, acute leukemia cases had similar proportions of birth terms (*p*-value = 0.891) and seasons of birth (*p*-value = 0.877).

For every one hundred meters closer that a mother resided to a major roadway at the time of delivery, the odds of having an offspring who developed ALL rose by 1% (aOR = 1.01; 95% CI: 1.00–1.02; Table 2). This result was consistent among children ≥1 to ≤5 years of age (aOR = 1.01; 95% CI: 1.00–1.02) and among children diagnosed at less than one year of age (aOR = 1.00; 95% CI: 0.97 1.04; Supplemental Table S1). For every one hundred meters closer that a mother resided to a major roadway at the time of delivery, no increase or decrease in odds of developing AML was evident (aOR = 1.00; 95% CI: 0.97 1.01). This estimate remained similar in subgroup analysis among children who developed AML between ≥1 and ≤5 years of age (aOR = 1.01; 95% CI: 0.97 1.04) and among those diagnosed at less than one year of age (aOR = 0.98; 95% CI: 0.95 1.00; Supplemental Table S1).

Overall, a strong association between maternal residence within 500 m of a major roadway and acute leukemia risk was not evident (Table 3). For example, mothers living within 500 m of a major roadway at the time of delivery were only 3% more likely to have an offspring who developed ALL compared to those living further than 500 m (aOR = 1.03; 95% CI: 0.91–1.16). In subgroup analysis among children ≥1 to ≤5 years of age, this association was slightly more apparent (aOR = 1.08; 95% CI: 0.94–1.24), although with uncertainty, as the 95% CI included the null. Among those diagnosed with ALL at less than one year of age, an inverse association was suggested (aOR: 0.79; 95% CI: 0.51–1.24; Supplemental Table S1). There was also no strong association apparent between maternal residence within 500 m of a major roadway and AML risk.

As with the other exposure metrics, roadway density was not strongly associated with the odds of having an offspring who would later develop ALL or AML (Table 4). Mothers residing in areas with medium roadway density (>0 km and <2.11 km of major roadways within the 500 m residential radius) were as likely to have an offspring who developed ALL as mothers living in areas with low roadway density (aOR = 1.00; 95% CI: 0.88–1.14). Mothers residing in high roadway density areas (≥2.11 km of major roadways within the 500 m residential radius) appeared to be 6% more likely to give birth to an offspring who developed ALL (aOR = 1.06; 95% CI: 0.92–1.20), although with uncertainty, since the 95% confidence interval included the null. In terms of ALL risk among those cases diagnosed at ≥1 to ≤5 years of age born to mothers who lived in areas with medium road density, a slight increase in risk was suggested (aOR = 1.07; 95% CI: 0.92–1.25). This observation was slightly more apparent for mothers residing in areas of high roadway density who gave birth to an offspring who developed ALL between the ages of ≥1 and ≤5 (aOR = 1.09; 95% CI: 0.93–1.28). Among those diagnosed at less than one year of age, modest inverse associations were suggested for both medium and high roadway densities in relation to ALL (Supplemental Table S1).

A strong effect was not evident between maternal residence in medium or high roadway density areas at the time of birth and AML (aOR = 0.86; 95% CI: 0.64–1.16, and aOR = 0.83; 95% CI: 0.61–1.13, respectively) among all ages. Among cases diagnosed between ≥1 and ≤5 years of age, no association was evident between maternal residence in medium roadway density areas and AML, although the effect estimate trended toward a positive association (aOR = 1.08; 95% CI: 0.70–1.67). Further, these younger AML cases were as likely to have a mother who resided in areas of high roadway density at time of birth as a mother who resided in low roadway density areas (aOR = 0.99; 95% CI: 0.64–1.55). Among those diagnosed at less than one year of age, modest inverse associations were suggested for both medium and high roadway densities in relation to AML (Supplemental Table S1).

## 4. Discussion

In this population-based case-control study, across all three proximity measures, modest effects at best were evident for both ALL and AML. In general, the association between major roadways and ALL risk was slightly stronger in children ages ≥1 to ≤5 years. However, effect estimates remained relatively close to the null suggesting that proximity to major roadways, as a proxy for measuring ambient air pollution, is not strongly associated with acute leukemia development. Among those diagnosed at less than one year of age, there was no association between distance to a major roadway and infant ALL or AML (ALL aOR = 1.00; 95% CI: 0.97–1.04 and AML aOR: 0.98; 95% CI: 0.95–1.00). When evaluating proximity and roadway density, inverse associations among those diagnosed under one year of age were suggested. Past studies have suggested that exposure to traffic-related air pollution may increase the risk of developing childhood malignancies such as acute leukemia [24,25,26,27]. Overall, research is inconclusive, and our results are consistent with several previous studies suggesting that traffic-related pollution may not strongly affect the odds of childhood cancer [13,52,53,54,55]. According to one of these studies, Janitz et al., found a modest, positive association between childhood acute leukemia and exposure to the fourth quartile of nitrogen dioxide (NO_2_; 11.19–19.89 ppb; aOR = 1.08; 95% CI: 0.75–1.55) [54]. In the current study, when the mother resided within 500 m of a major roadway at the time of birth, there was no observed trend between living in high roadway density areas and the development of acute leukemia (aOR = 1.02; 95% CI: 0.90–1.15) compared to mothers living in low roadway density areas. Similarly, Houot et al., found that when children lived <150 m of a major roadway at the time of diagnosis, there was no strong increase in risk of ALL or AML (OR = 1.0; 95% CI: 0.9–1.1, and OR = 1.2; 95% CI: 0.9–1.5, respectively) [55]. In a study conducted by Raaschou-Nielsen et al., no elevated risk for a child developing acute leukemia was evident if the mother resided in traffic areas with an average of ≥10,000 vehicles per day during pregnancy (OR = 0.8; 95% CI: 0.5–1.3) compared to children whose mothers resided in traffic areas with an average of <500 vehicles per day [52].

The current study utilized maternal residence at the time of birth as a proxy measure to evaluate exposure to air pollution. It is established that traffic-related pollution contains harmful particulate matter and identified human carcinogens. For instance, exposure to particulate air pollution is known to contain free radicals and cause oxidative stress which facilitates DNA damage and can trigger mutagenesis [56,57]. Furthermore, maternal exposure to high levels of polycyclic aromatic hydrocarbons (PAHs), carcinogenic compounds found in motor vehicle exhaust, is linked to fetal PAH–DNA adduct formation and can increase the risk of the offspring developing cancer later in life [58,59]. Moreover, aromatic hydrocarbons like benzene have been classified by the International Agency for Research on Cancer (IARC) as carcinogenic to humans, giving rise to AML and possibly the risk of ALL [11,60]. Due to these negative health implications of traffic-related air pollution, we sought to determine if exposure to traffic-related pollution affects the odds of developing childhood acute leukemia.

These results should be considered in light of certain limitations. For example, this study relied on maternal proximity to major roadways as a substitute measure for determining air pollution exposure. This type of measurement was applied as it is more difficult and costly to use other methods to obtain personal exposure levels to air pollutants. Moreover, air monitors are placed inconsistently across Texas and may not necessarily yield accurate results when compared to the use of proximity measures, which have been shown to be an adequate proxy for estimating air pollution including particulate matter (e.g., NO_2_) [61]. Another limitation is that residential mobility could lead to exposure misclassification. However, recent studies suggest that individuals tend to move short distances into areas with similar air pollution profiles [62,63,64]. Over 95% of both case and control mothers in this study resided in urban areas at time of delivery. Therefore, our results may reflect urban exposure levels. While there is variability in air pollution levels across urban areas, our model may not have adequately captured that. However, as this is a population-based study, we obtained cases and controls from across the entire state of Texas, which should help to lessen any potential bias related to this. Another limitation is that as the analyzed data were based on birth records, our investigations were constrained to within this data source. Specifically, we were unable to investigate how observed associations would be modulated if other strong risk predictors of acute leukemia and markers of childhood leukemia heterogeneity, such as cytogenetic abnormalities [65,66], were taken into account. We were also unable to assess the impact of other maternal health behaviors that might influence risk, or how other potentially confounding factors (e.g., environmental risk factors) may have influenced the reported results. It is important to note that while these limitations relate to our use of vital statistics, birth statistics in combination with cancer registry data have been leveraged to inform how maternal and perinatal characteristics impact the risk of several childhood cancers [67,68,69,70]. Another limitation is that we received the geocoding data for this study from the State Health Services in Texas. Due to data suppression rules, data could only be evaluated for buffers of within 500 meters to a residence to limit the potential for subject identification. Therefore, we were unable to assess shorter exposure distances in this study. However, there is support for analyses investigating 500 m buffers as done herein [19,31,71]. Future studies should put planning in place to consider smaller buffer regions.

This study has several important strengths. One is the use of a population-based design that reduced the potential effects of participation bias. The use of birth records to identify cases and controls helped to ensure that the population of interest was well represented [72]. Information from birth certificates has also been shown to be a reliable source of data [73,74]. Moreover, this study contains 16 years of cancer incidence data drawn from one of the largest statewide cancer registries, in a state characterized by variable and high levels of traffic-related air pollution and roadway densities. Coupled with the large sample size of over 2000 acute leukemia cases, which to our knowledge is one of the largest studies conducted in Texas on the relationship between maternal proximity to major roadways and childhood leukemia risk, this study was adequately powered to assess the impact of traffic-related air pollution on the odds of developing childhood acute leukemia. A further strength of these analyses is that several measures of maternal proximity to major roadways were assessed (continuous distance to the nearest roadway, if the mother resided within 500 m of a major roadway, and roadway density), and results across all three measures remained relatively consistent. This analysis also focused on childhood acute leukemia, which tends to have shorter latency periods and fewer methodological obstacles for this type of study [75]. The ability to focus on children allowed for suitable exposure measurement as measurement at the time of delivery is generally considered relevant for other early life exposures, such as at the time of conception [64] and early childhood [76].

## 5. Conclusions

This large population-based assessment suggests that a modest effect exists between mothers residing close (<500 m) to a major roadway or in high roadway density areas and the odds of giving birth to a child who will develop ALL or AML. These results will inform future research studying this relationship as increasing segments of the U.S. population live in or near major metropolitan areas with dense networks of major roadways. Upcoming studies should utilize novel methods to directly measure exposure, such as through biomarkers of traffic-related air pollution exposure, and evaluate the role of other potential genetic and environmental factors in the development of acute leukemia.

## Figures and Tables

**Table 1:** Demographic characteristics of cases with acute leukemia and birth certificate controls in Texas, 1995–2011.

Characteristics	Any Leukemia (*n* = 2030)	ALL (*n* = 1746)	AML (*n* = 284)	Controls (*n* = 20,300)	*p*-Value
**Child**					
Child’s sex, *n* (%)					0.001
Male	1114 (54.9)	958 (54.9)	156 (54.9)	10,258 (50.5)	
Female	916 (45.1)	788 (45.1)	128 (45.1)	10,042 (49.5)	
Age at diagnosis (years), *n* (%)					
<1	180 (8.9)	105 (6.0)	75 (26.4)		
≥1 to ≤5	1377 (67.8)	1232 (70.6)	145 (51.1)		
>5 to ≤10	368 (18.1)	329 (18.8)	39 (13.7)		
>10	105 (5.2)	80 (4.6)	25 (8.9)		
Season of birth, *n* (%)					0.88
Summer (June–August)	535 (26.4)	457 (26.2)	78 (27.5)	5361 (26.4)	
Fall (September–November)	520 (25.6)	444 (25.4)	76 (26.8)	5228 (25.8)	
Winter (December–February)	506 (25.0)	429 (24.6)	77 (27.1)	4908 (24.2)	
Spring (March–May)	469 (23.1)	416 (23.8)	53 (18.7)	4803 (23.7)	
Term of birth, *n* (%)					0.89
<37 weeks	240 (11.8)	192 (11.0)	48 (16.9)	2351 (11.6)	
37 to <41 weeks	1553 (76.5)	1344 (77.0)	209 (73.6)	15,525 (76.5)	
41 to <42 weeks	156 (7.7)	140 (8.0)	16 (5.6)	1654 (8.2)	
42 weeks to <44 weeks	52 (2.7)	42 (2.4)	10 (3.5)	504 (2.5)	
Unknown	29 (1.4)	28 (1.6)	1 (0.35)	266 (1.3)	
Gestational size, *n* (%)					<0.001
Small	239 (11.8)	199 (11.4)	40 (14.1)	2778 (13.7)	
Appropriate	1532 (75.5)	1316 (75.4)	216 (76.1)	15,601 (76.9)	
Large	236 (11.6)	209 (12.0)	27 (9.5)	1700 (8.4)	
Unknown	23 (1.1)	22 (1.3)	1 (0.4)	213 (1.1)	
***Maternal***					
Maternal race/ethnicity, *n* (%)					<0.001
non-Hispanic white	731 (36.0)	627 (35.9)	104 (36.6)	8118 (40.0)	
non-Hispanic black	135 (6.7)	102 (5.8)	33 (11.6)	2595 (12.8)	
Hispanic	1085 (53.5)	952 (54.5)	133 (46.8)	8772 (43.2)	
non-Hispanic other	79 (3.9)	65 (3.7)	14 (4.9)	815 (4.0)	
Age at delivery (years), *n* (%)					<0.001
<25	766 (37.7)	663 (38.0)	103 (36.3)	8488 (41.8)	
25–30	568 (28.0)	487 (27.9)	81 (28.5)	5565 (27.4)	
31–35	408 (20.1)	361 (20.7)	47 (16.6)	4083 (20.1)	
≥35	288 (14.2)	235 (13.5)	53 (18.7)	2164 (10.7)	
Education, *n* (%)					0.001
<High School	682 (33.6)	586 (33.6)	96 (33.8)	6041 (29.8)	
Completed High School	547 (27.0)	477 (27.3)	70 (24.7)	5959 (29.4)	
≥High School	778 (38.3)	662 (37.9)	116 (40.9)	8100 (39.9)	
Unknown	23 (1.1)	21 (1.2)	2 (0.7)	200 (1.0)	
**Neighborhood at birth**					
Urban status, *n* (%)					0.96
Urban	1944 (95.8)	1676 (96.0)	268 (94.4)	19,435 (95.7)	
Rural	86 (4.2)	70 (4.0)	16 (5.6)	865 (4.3)	
Area-Level poverty, *n* (%)					0.06
<15% of households	1068 (52.6)	920 (52.7)	148 (52.1)	11,121 (54.8)	
≥15% of households	962 (47.4)	826 (47.3)	136 (47.9)	9179 (45.2)	
**Maternal proximity to major roads**					
Continuous distance (m), mean (SD)	389.7 (523.5)	383.0 (506.5)	430.8 (617.7)	404.0 (574.2)	0.28
Within 500 m, *n* (%)					0.81
Exposed	1551 (76.4)	1342 (76.9)	209 (73.6)	15,462 (76.2)	
Unexposed	479 (23.6)	404 (23.1)	75 (26.4)	4838 (23.8)	
Roadway Density, *n* (%) ^a^					0.74
Low	479 (23.6)	404 (23.1)	75 (26.4)	4838 (23.8)	
Medium	764 (37.6)	658 (37.7)	106 (37.3)	7767 (38.3)	
High	787 (38.8)	684 (39.2)	103 (36.3)	7695 (37.9)	

*n*, number of cases or controls; ALL, acute lymphoblastic leukemia; AML, acute myeloid leukemia; km, kilometer; SD, standard deviation; m, meter. ^a^ Low, residence >500 m from nearest major roadway; Medium, >0 and <1.53 km of major roadways within 500 m residential radius; High, ≥1.53 km of major roadways within 500 m residential radius.

**Table 2:** Associations between continuous distance of maternal residence to nearest major roadway and leukemia in offspring.

			All Ages		Ages ≥1 to ≤5			
Leukemia Subtype	Cases Mean (SD)	Controls Mean (SD)	OR ^a^ (95% CI) ^b^	aOR (95% CI) ^c^	Cases Mean (SD)	Controls Mean (SD)	OR (95% CI) ^b^	aOR (95% CI) ^c^
ALL	383.0 m (506.5)	404.0 m (574.2)	1.01 (1.00–1.02)	1.01 (1.00–1.02)	398.4 m (536.3)	404.0 m (574.2)	1.00 (1.00–1.01)	1.01 (1.00–1.02)
AML	430.8 m (617.7)	404.0 m (574.2)	1.00 (0.98–1.01)	1.00 (0.97–1.01)	386.6 m (569.4)	404.0 m (574.2)	1.01 (0.98–1.04)	1.01 (0.97–1.04)

ALL, acute lymphoblastic leukemia; AML, acute myeloid leukemia; SD, standard deviation; OR, odds ratio; CI, confidence interval; aOR, adjusted odds ratio. ^a^ Table 2 ORs and aORs reflect estimates per 100 m; ^b^ adjusted for birth year; ^c^ adjusted for birth year, maternal race/ethnicity (categorical), maternal education (categorical), area-level poverty (categorical), birth weight in grams (continuous), and child sex (categorical).

**Table 3:** Associations between maternal residential proximity within 500 m of a major roadway and leukemia in offspring.

			All Ages		Ages ≥1 to ≤5			
Proximity to Major Roadway	Cases *n (%*)	Controls *n (%*)	OR (95% CI) ^a^	aOR (95% CI) ^b^	Cases *n (%*)	Controls *n (%*)	OR (95% CI) ^a^	aOR (95% CI) ^b^
**ALL**								
>500 m	404 (23.1)	4838 (23.8)	Reference (1.00)	Reference (1.00)	286 (23.2)	4838 (23.8)	Reference (1.00)	Reference (1.00)
≤500 m	1342 (76.9)	15,462 (76.2)	1.04 (0.92–1.17)	1.03 (0.91–1.16)	946 (76.8)	15,462 (76.2)	1.05 (0.92–1.21)	1.08 (0.94–1.24)
**AML**								
>500 m	75 (26.4)	4838 (23.8)	Reference (1.00)	Reference (1.00)	33 (22.8)	4838 (23.8)	Reference (1.00)	Reference (1.00)
≤500 m	209 (73.6)	15,462 (76.2)	0.88 (0.68–1.15)	0.84 (0.64–1.11)	112 (77.2)	15,462 (76.2)	1.08 (0.73–1.60)	1.04 (0.70–1.55)

ALL, acute lymphoblastic leukemia; AML, acute myeloid leukemia; m, meter; *n*, Number of cases or controls; OR, odds ratio; CI, confidence interval; aOR, adjusted odds ratio. ^a^ adjusted for birth year; ^b^ adjusted for birth year, maternal race/ethnicity (categorical), maternal education (categorical), area-level poverty (categorical), birth weight in grams (continuous), and child sex (categorical).

**Table 4:** Associations between maternal residential roadway density and leukemia in offspring.

			All Ages		Ages ≥1 to ≤5			
Roadway Density	Cases *n (%*)	Controls *n (%*)	OR (95% CI) ^a^	aOR (95% CI) ^b^	Cases *n (%*)	Controls *n (%*)	OR (95% CI) ^a^	aOR (95% CI) ^b^
**ALL**								
Low	404 (23.1)	4838 (23.8)	Reference (1.00)	Reference (1.00)	286 (23.2)	4838 (23.8)	Reference (1.00)	Reference (1.00)
Medium	658 (37.7)	7767 (38.3)	1.01 (0.89–1.15)	1.00 (0.88–1.14)	476 (38.6)	7767 (38.3)	1.05 (0.90–1.22)	1.07 (0.92–1.25)
High	684 (39.2)	7695 (37.9)	1.06 (0.93–1.21)	1.06 (0.92–1.20)	470 (38.2)	7695 (37.9)	1.06 (0.91–1.23)	1.09 (0.93–1.28)
**AML**								
Low	75 (26.4)	4838 (23.8)	Reference (1.00)	Reference (1.00)	33 (22.8)	4838 (23.8)	Reference (1.00)	Reference (1.00)
Medium	106 (37.3)	7767 (38.3)	0.89 (0.66–1.20)	0.86 (0.64–1.16)	59 (40.7)	7767 (38.3)	1.13 (0.74–1.74)	1.08 (0.70–1.67)
High	103 (36.3)	7695 (37.9)	0.87 (0.65–1.18)	0.83 (0.61–1.13)	53 (36.6)	7695 (37.9)	1.03 (0.67–1.60)	0.99 (0.64–1.55)

ALL, acute lymphoblastic leukemia; AML, acute myeloid leukemia; *n*, number of cases or controls; OR, odds ratio; CI, confidence interval; aOR, adjusted odds ratio. ^a^ Adjusted for birth year; ^b^ adjusted for birth year, maternal race/ethnicity (categorical), maternal education (categorical), area-level poverty (categorical), and birth weight in grams (continuous) and child sex (categorical).

## Supplementary Materials

The following are available online at https://www.mdpi.com/1660-4601/16/11/2029/s1, Table S1: Associations between Proximity of Maternal Residence to Nearest Major Roadway and Leukemia in Offspring Diagnosed Under One Year of Age.
