# Supplementary material for: Maternal Residential Proximity to Major Roadways and the Risk of Childhood Acute Leukemia: A Population-Based Case-Control Study in Texas, 1995–2011
Source: Int J Environ Res Public Health. 2019 Jun 7;16(11):2029. doi: 10.3390/ijerph16112029 (PMC6603856; doi:10.3390/ijerph16112029)
Supplement: Supplementary file 1 [file ijerph-16-02029-s001.pdf]

Supplemental Table S1. Associations between Proximity of Maternal Residence to Nearest Major Roadway and Leukemia in Offspring Diagnosed Under One Year of Age.

			Less than One Year of Age	
**Continuous Distance**	Cases<br>*Mean (SD)*	Controls<br>*Mean (SD)*	OR [a]<br>(95% CI) [b]	aOR<br>(95% CI) [c]
ALL	383.8 m<br>(469.0)	404.0 m<br>(574.2)	1.01<br>(0.97-1.05)	1.00<br>(0.97-1.04)
AML	543.3 m<br>(788.7)	404.0 m<br>(574.2)	0.98<br>(0.95-1.01)	0.98<br>(0.95-1.00)
**Proximity to Major Roadway**	Cases<br>*n* (%)	Controls<br>*n* (%)	OR<br>(95% CI) [b]	aOR<br>(95% CI) [c]
ALL				
> 500 m	28<br>(26.7)	4838<br>(23.8)	Reference<br>(1.00)	Reference<br>(1.00)
≤ 500 m	77<br>(73.3)	15,462<br>(76.2)	0.90<br>(0.58-1.39)	0.79<br>(0.51-1.24)
AML				
> 500 m	23<br>(30.7)	4,838<br>(23.8)	Reference<br>(1.00)	Reference<br>(1.00)
≤ 500 m	52<br>(69.3)	15,462<br>(76.2)	0.77<br>(0.47-1.25)	0.74<br>(0.45-1.22)
**Roadway Density**	Cases<br>*n* (%)	Controls<br>*n* (%)	OR<br>(95% CI) [b]	aOR<br>(95% CI) [c]
ALL				
Low	28<br>(26.7)	4,838<br>(23.8)	Reference<br>(1.00)	Reference<br>(1.00)
Medium	38<br>(36.2)	7,767<br>(38.3)	0.88<br>(0.54-1.44)	0.80<br>(0.49-1.31)
High	39<br>(37.1)	7,695<br>(37.9)	0.93<br>(0.57-1.51)	0.79<br>(0.48-1.30)
AML				
Low	23<br>(30.7)	4,838<br>(23.8)	Reference<br>(1.00)	Reference<br>(1.00)
Medium	24<br>(32.0)	7,767<br>(38.3)	0.70<br>(0.39-1.23)	0.69<br>(0.39-1.23)
High	28<br>(37.3)	7,695<br>(37.9)	0.84<br>(0.48-1.46)	0.79<br>(0.45-1.40)

ALL, acute lymphoblastic leukemia; AML, acute myeloid leukemia; SD, standard deviation; OR, odds ratio; CI, confidence interval; aOR, adjusted odds ratio; *n*, Number of cases or controls. [a] Continuous distance ORs and aORs reflect estimates per 100 m; [b] Adjusted for birth year; [c] Adjusted for birth year, maternal race/ethnicity (categorical), maternal education (categorical), area-level poverty (categorical), birth weight in grams (continuous) and child sex (categorical).
